# Honoring the Care Experiences of Chinese Canadian Survivors of Prostate Cancer to Cultivate Cultural Safety and Relationality in Digital Health: Exploratory-Descriptive Qualitative Study

**DOI:** 10.2196/49349

**Published:** 2023-12-28

**Authors:** Karen Young, Ting Xiong, Rachel Lee, Ananya Tina Banerjee, Myles Leslie, Wellam Yu Ko, Julia Yu Jia Guo, Quynh Pham

**Affiliations:** 1 Centre for Digital Therapeutics Techna Institute University Health Network Toronto, ON Canada; 2 Institute of Health Policy, Management and Evaluation Dalla Lana School of Public Health University of Toronto Toronto, ON Canada; 3 Department of Epidemiology, Biostatistics and Occupational Health McGill University Montreal, QC Canada; 4 School of Public Policy University of Calgary Calgary, AB Canada; 5 Men’s Health Research Program University of British Columbia Vancouver, BC Canada; 6 Toronto General Hospital Research Institute University Health Network Toronto, ON Canada; 7 Telfer School of Management University of Ottawa Ottawa, ON Canada; 8 School of Public Health Sciences University of Waterloo Waterloo, ON Canada

**Keywords:** digital health, virtual care, digital therapeutics, prostate cancer, cancer survivorship, social determinants of health, structural determinants of health, supportive care, cultural adaptation, Chinese Canadians

## Abstract

**Background:**

Prostate cancer (PCa) is the most commonly diagnosed nonskin cancer for Canadian men and has one of the highest 5-year survival rates, straining systems to provide care. Virtual care can be one way to relieve this strain, but survivors’ care needs and technology use are influenced by intersecting social and cultural structures. Cultural adaptation has been posited as an effective method to tailor existing interventions to better serve racialized communities, including Chinese men. However, cultural adaptations may inadvertently draw attention away from addressing structural inequities.

**Objective:**

This study used qualitative methods to (1) explore the perceptions and experiences of Chinese Canadian PCa survivors with follow-up and virtual care, and (2) identify implications for the cultural adaptation of a PCa follow-up care app, the *Ned* (no evidence of disease) Clinic.

**Methods:**

An axiology of relational accountability and a relational paradigm underpinned our phenomenologically informed exploratory-descriptive qualitative study design. A community-based participatory approach was used, informed by cultural safety and user-centered design principles, to invite Chinese Canadian PCa survivors and their caregivers to share their stories. Data were inductively analyzed to explore their unmet needs, common experiences, and levels of digital literacy.

**Results:**

Unmet needs and technology preferences were similar to broader trends within the wider community of PCa survivors. However, participants indicated that they felt uncomfortable, unable to, or ignored when expressing their needs. Responses spoke to a sense of isolation and reflected a reliance on culturally informed coping mechanisms, such as “eating bitterness,” and familial assistance to overcome systemic barriers and gaps in care. Moreover, virtual care was viewed as “better than nothing;” it did not change a perceived lack of focus on improving quality of life or care continuity in survivorship care. Systemic changes were identified as likely to be more effective in improving care delivery and well-being rather than the cultural adaptation of *Ned* for Chinese Canadians. Participants’ desires for care reflected accessibility issues that were not culturally specific to Chinese Canadians.

**Conclusions:**

Chinese Canadian survivors are seeking to strengthen their connections in a health care system that provides privacy and accessibility, protects relationality, and promotes transparency, accountability, and responsibility. Designing “trickle-up” adaptations that address structural inequities and emphasize accessibility, relationality, and privacy may be more effective and efficient at improving care than creating cultural adaptations of interventions.

## Introduction

Approximately 1 in 8 Canadian men will be diagnosed with prostate cancer (PCa), and the overall 5-year survival rate has reached 91% [1]. Canada has experienced a growing clinical and economic burden associated with PCa survivorship (or follow-up) care, while survivors continue to experience unmet needs [2-4]. Virtual care and digital health, which refer to remotely delivered care via information technology, can be one pathway to relieving resource constraints while facilitating holistic and inclusive survivorship care delivery [5,6]. Further, the increasing proliferation of virtual care services has made it unlikely that care will fully return to in-person delivery in Canada [7,8]. A review of virtual cancer survivorship models indicated that remote delivery of survivorship care through genitourinary telemedicine clinics and a PCa surveillance program resulted in safe and cost-effective delivery of care, accompanied by high patient satisfaction [5]. To leverage the benefits of digital health for PCa follow-up care, the *Ned* (no evidence of disease) Clinic was designed to allow patients to report their outcomes (patient-reported outcomes) through the *Ned* app, while clinicians virtually provide care [9]. It was developed by a consortium of stakeholders in Ontario, Canada, in adherence to endorsed practice guidelines.

However, PCa survivors’ outcomes and needs are affected by complex and intersecting structural and social determinants of health (SDOH), such as race, gender, culture, income, public policy, institutional practice, and more [10-13]. Culturally adapting existing interventions is posited to address these factors, leveraging their benefits and saving resources via changes based on “surface” (social and behavioral characteristics) and “deep” (worldview, norms, beliefs, and values) cultural structures [14]. Meta-analyses indicate that culturally adapted interventions are more effective for specific communities [15]. While cultural adaptations of PCa surveillance tools exist, follow-up care protocols and cancer survivorship virtual care apps do not appear to have been previously culturally adapted. This lack of evidence is likely owing to the nascency of virtual survivorship care [5].

Concurrently, digital technology has been shown to exacerbate inequities [14]. The digital determinants of health (DDOH), like their structural and social counterparts (SDOHs), have resulted in a “digital divide” between those with digital access and literacy and those without [14]. For example, digitizing health services can prevent these services from being accessed by those without regular internet availability, which can be the result of financial or geographic constraints (an SDOH) [15]. These determinants have been created within the larger backdrop of settler colonialism in Canada [16]. Settler colonialism requires the colonizer to settle on and extract resources from land via chattel slavery and indentured labor. In Canada, it has resulted in the centuries-long genocide and dispossession of Indigenous communities who lived in relation to these lands and discriminatory practices against nonwhite communities [1,12].

The experiences of Chinese Canadian PCa survivors illustrate these complex intersections. Chinese Canadians are the second-largest “visible minority” group in Canada, but are less likely to access care and more likely to report lower satisfaction when they do [16,17]. Race, an SDOH, has been linked to variable overall survival and cancer-specific survival rates [18]. Although Asian men, including ethnic Chinese men, have better overall survival and cancer-specific survival than the median, they are also more likely to present with advanced PCa [19]. Asian and Pacific Islander communities are often grouped together in race-based analyses of cancer survivorship data. However, disaggregating analyses show significant variations among ethnic groups within this categorization [19,20]. This indicates that more careful disaggregation of data and attention to sociodemographic factors that affect treatment and care are needed. Indeed, previous work on the experiences of Chinese Canadians with PCa survivorship or virtual care has not been published.

In consideration of these factors, we sought to uncover the social structures that may influence the perception of care and technology among Chinese Canadian PCa survivors. Of particular interest is the role of Canada’s settler colonial structures in their experiences [21-23]. These structures underpin notions of technology, identity, health, and culture as imagined and practiced in Canada. They are the foundations of hegemonic beliefs, such as the model minority stereotype, which has resulted in inadequate health care provision, improper health data aggregation, and the racist treatment of Asian individuals in care settings [19,24,25].

With this context, it is increasingly important to create digital health innovations that provide safe care and accurate information. These evidence gaps regarding Chinese Canadians’ experiences with PCa survivorship, virtual care, and the overall gap regarding cultural adaptation of virtual survivorship care models necessitated an approach from the ground up. Using qualitative methods, the purpose of this study was to (1) explore the perceptions and experiences of Chinese Canadian PCa survivors with follow-up and virtual care, and (2) within these experiences, identify patterns relating to social structures shaping user needs within this community. Together, this work aims to illustrate the downstream effects of social structures on the virtual care and follow-up care experiences of these survivors, while surfacing their desires regarding virtual PCa follow-up care to adapt the patient-facing *Ned* Clinic app.

## Methods

### Study Overview

This phenomenologically informed exploratory-descriptive qualitative study was designed as the first phase of a larger research project to investigate options for culturally adapting the *Ned* Clinic for Chinese Canadian PCa survivors [26,27]. This study was conducted at the University of Toronto between September and December 2022 during the COVID-19 pandemic [26,28]. Data collection and analysis were completed by KY, RL, JYJG, and TX. Multiple strategies were used to ensure rigor, such as appropriate frameworks, rich descriptions of the context and sample, and theoretically informed methods [29]. Reflexivity was incorporated by applying *Etuaptmumk*, or two-eyed seeing, cultural safety, and intersectionality throughout the project [30-32]. We present this study according to the Consolidated Criteria for Reporting Qualitative Research (COREQ) guidelines [33].

### Ethical Considerations

Research ethics approval was obtained from the University of Toronto Research Ethics Board (human protocol #43145).

### Research Paradigm

To operationalize a stance of resistance against settler colonialism, we turned to Indigenous and Black feminist ontologies, epistemologies, and frameworks for guidance. First, to denaturalize settler colonialism, we redefined “immigrants” as people with ancestral roots outside of Indigenous lands, who are beholden to Indigenous laws and epistemologies [21]. We grounded ourselves in knowing that decolonization is always “only accountable to Indigenous sovereignty and futurity” [21]. Immigrants cannot become Indigenous as one might become a Canadian, but similar experiences of oppression can allow for strategic collaboration.

Different paradigmatic elements can be interwoven if the axiomatic reasoning is strongly resonant [34]. This project was first rooted in an axiology of relational accountability [35]. We sought to gain knowledge for the purpose of fulfilling our obligations to do things in a good way with our relations [36]. Thus, this axiology impelled our perception of relationships as the “building blocks” of our realities. These relations define the knowledges we have and how it can be learned or transmitted. Black feminist thought, a field of critical theory, views lived experience to be inextricably linked with unbalanced social structures (ie, the SDOH) [37]. However, lived experience alone is a partial truth, as it is the value of the collective that provides fuller evidence of these structures. Epistemologically, the field premises knowledge via “the knower” and “collective knowers” [37]. The knower is the individual’s lived, specific experience. The collective knowers are where knowledge is shared to expose these structures. The interweaving of these elements was necessitated by the particularly anti-Indigenous and anti-Black structures of settler colonialism.

By applying this paradigm and following the paths of Indigenous women who have indigenized digital spaces for Indigenous resurgence, we regrounded our understanding of the digital as landed and limited, rather than invisible and ubiquitous [38]. These land relations should shape the design and development of any digital app. Then, we oriented ourselves with the Mi’kmaq guiding principle *Etuaptmumk*, or two-eyed seeing, to develop a multilevel, holistic approach that acknowledges the multiplicity and diversity of knowledge-systems, while appreciating that humility is a necessary ingredient because knowledge always changes [30,39].

### Frameworks

Our relational grounding called for a community-based participatory research (CBPR) approach. For communities that face barriers to care, it was found that CBPR practices are appropriate, such as our engagement of a key informant and invitations to community members to share their lived experiences through open-ended interviews [7,40]. Cultural safety principles were applied to prioritize the participants’ safety throughout the research process [31]. Intersectionality was used as a prism to identify how similar strategies of discrimination have differential effects [32]. A desire-based approach encouraged participants to engage the whole of their personhood to dream futurities involving PCa survivorship care and virtual care [36,41]. Finally, user-centered design (UCD) concepts were used to formalize themes important to end users [42].

### Setting and Place

This study was conducted in the Greater Vancouver Regional District (GVRD), located on the current, unceded, and future territories of the šxʷməθkʷəy̓əmaɁɬ təməxʷ, Skwxwú7mesh-ulh Temíx̱w, and səl̓ilwətaɁɬ təməxʷ (Tsleil-Waututh, Squamish, and Musqueam, respectively) First Nations. The GVRD is home to one of Canada’s largest, oldest, living Chinese communities, including persons and families whose arrivals, lives, and settlement in Canada span multiple geographies and generations [43]. The community’s continuation is made possible by continuing Indigenous genocide and land dispossession as a result of settler colonialism. The experiences described here were shaped and materialized in the presence of this history, the continued settlement of these lands, and the health care systems that were established there. We note that the GVRD holds a long history of anti-Asian racism and discrimination that has and continues to affect the lives, livelihoods, and health care of Chinese people in this area for centuries [44-46].

### Participants

We recruited survivors who (1) lived in and received PCa follow-up care in Canada (or were partnered to someone who did); (2) self-identified as Chinese or Chinese Canadian; and (3) were fluent in English, Cantonese, or Mandarin for enrollment. As partners play a large role in survivorship care, we also recruited partner-caregivers (PCs) for this study [47,48]. All PCs were snowball sampled from participating survivors. We use community to represent a “symbolic totality as well as a practical multiplicity” [49]. The establishment of this community and its shape are a result of the GVRD’s geography. We address our participants as a coalition of self-identified Chinese Canadians impacted by PCa survivorship to attend to the differential and pragmatic nature of its formation.

The lead (KY) and senior author (QP) established trusted relations with a supportive care program that provides care for Chinese Canadian PCa survivors and a Chinese PCa support group in this area. A key community informant agreed to guide this study and review and approve study materials. Group administrators were briefed, and study invitations were issued to members through a convenience sampling strategy. Eligible members were asked to consent to participate; verbal and written consent was obtained. Phenomenological concepts informed data collection to account for the multidimensionality of lived experience, survivors’ embodied feelings of safety, and possible hurdles to reaching saturation [27,50].

### Interviews and Data Collection

The semistructured interview guides were based on Tuck’s [41] desire-based approach, Spanhel et al's cultural adaptation taxonomy [15], phenomenological inquiry, and UCD to invite participants to explore the benefits, desires, and challenges that they had in accessing survivorship care and virtual care [42,51]. Please refer to Multimedia Appendix 1 and Multimedia Appendix 2 for the full survivor and PC interview guides. Prior to data collection, interviewers completed training in antioppression and cultural safety. Participants were offered an interview in Mandarin, Cantonese, or English by videoconference (Microsoft Teams or Zoom [Zoom Video Communications, Qumu Corporation]) or telephone because of the COVID-19 pandemic and gave consent in their language of choice. Interviewers created reflexive field notes after each session. Sessions were audio-recorded with participants’ consent for transcription and translation. The translation of interview materials and outputs into English broadly followed methods outlined in Haldane et al [52]. Forms were completed through the REDCap (Research Electronic Data Capture; Vanderbilt University) tool at the University of Toronto [53].

### Data Analysis

Descriptive statistics were compiled from demographic forms submitted by participants. Data analysis occurred both contemporaneously with, and subsequent to, data collection, and followed an inductive thematic analytic approach [54]. 2 coders, KY and TX, used NVivo (QSR International) software to code all the transcripts individually. First, the coders read through all transcripts to “get a sense of the whole,” then jointly compiled an initial codebook [55]. This codebook was iteratively refined to improve reasoning via negotiated agreement between the coders [56]. Once codebook agreement was reached, the remaining transcripts were separately coded and used to continue refining the codebook, from which a set of themes was distilled.

### Positionality

A key marker of excellent qualitative research is positionality, which indicates that the researcher is reflective and aware of their values, experiences, biases, and inclinations within their research [29]. We report on their social positions, personal experiences, and political and professional beliefs here because these may all affect the manner in which the research problem was framed and defined, the methods used, and the reporting of the results [57].

KY is a health informatics trainee and second-generation Chinese Canadian settler who was born and raised in the GVRD by a working-class, first-generation immigrant family with roots in southeastern China. She grew up with Chinese and Canadian cultural traditions but has identified more closely with Western ontologies and epistemologies because of her education in settler colonial settings. KY has acted in capacity as a caregiver, health care intermediary, and translator for multiple family members of both genders in the Canadian health care system. She does not have any direct experience with PCa and has not previously provided care for PCa survivors.

TX is a first-generation Chinese Canadian and a recent immigrant who was born in a rural setting in southeastern China. She transitioned from a rural area to an urban setting while growing up, then immigrated from China to Canada as a young adult. TX is more familiar with Chinese cultural beliefs than Canadian traditions. She has not directly cared for anyone who lived or is living with PCa and therefore cannot fully comprehend the challenges PCa survivors may experience. TX has trained in health services and health informatics, completed research on PCa follow-up care, and is familiar with cancer survivorship virtual care models.

## Results

### Overview

Participants’ responses reflected a reliance on culturally informed coping mechanisms and familial assistance to manage a lack of support and understanding provided by the health care system in processing their PCa experiences, systemic patient-provider communication barriers, and perceived breakdowns in health care access. Experiences reflected an awareness that virtual care provided convenience but could also fracture tenuous patient-provider-system relationships that would result in the further isolation of survivors.

### Demographics

A total of 14 participants were interviewed to reach saturation. The average age of participants was 66 (SD 8.43) years. All survivors identified as men (n=12, 86%) and all PCs as women (n=2, 14%). Further, 13 (93%) participants indicated that they spoke English as an additional language (EAL). Most made an income between CAD $15,000 and CAD $100,000 (US $11,048 and US $73,653; n=12, 86%), lived in an urban area (n=13, 93%), were married (n=12, 86%), and were educated beyond high school (n=13, 93%). A majority were retired (n=9, 64%), but some continued in paid employment. A equal split emerged between preferences for smartphone or desktop or laptop use. Most (n=10, 71%) self-rated as comfortable with their device. Participants indicated that they had 2 or fewer smartphone health apps (n=13, 36%). For a detailed breakdown, please refer to Multimedia Appendix 3.

### “Close to Their Heart”: the Hidden Emotional Impact of PCa

Participants spoke of feelings of isolation and an overall lack of social support, emotional outlets, and coping strategies when referring to the impact of PCa. The follow-up care they received did not appear to adequately address the mental or emotional aspects of PCa. Participants continued to feel heavy emotions when they thought of their experiences with their disease, providers, or the health care system. They actively tried to minimize and avoid reliving these emotions and experiences, but nevertheless continued to struggle with them, even long after they finished treatment. Ethnicity was implicated by some when referring to this norm:

I don’t know about others but I can speak for the Chinese. The Chinese will hold those things very close to their heart. It’s not something that they would want to advertise. You know it’s almost like it’s their fault that they got this problem.

Participant S007

Within this broader norm of shame and the pressure to hide, some expressed that they were uncomfortable even speaking of their diagnosis at all, as they felt that sharing would be burdensome. Those who shared their diagnosis did so out of a desire to assist other people with PCa, but they refrained from speaking about their emotions. A select few who shared their feelings did so only with very close relations. Some indicated that this study was the first time they felt comfortable disclosing their emotions, as we had created a safe, private environment.

Although a few spoke to the need for better mental health support, this was proposed for the community at large rather than as an individual need. This private endurance of hardship reflected patterns of shame and individualized responsibility for larger systemic problems as coping mechanisms. Participants’ stories indicated that if their care requests were rebuffed, they would endure the difficulty or attempt to resolve it through privately soliciting support from family or friends. Those who passively accepted their disease progression and treatment outcomes appeared to process these events as a matter of fate. By adopting fatalism, they implied acceptance of their disease status and the possibility of death as a result of their PCa.

I don’t really go chasing information, not that type of person, that might be the reason. Most of the time, in Cantonese, “you eat what you have been cooked.” Whatever comes, comes, you know? Whatever will be, will be. So you don’t pay attention to it.

Participant S007

We posit that these norms hindered our initial snowball sampling strategy. In total, 2 PCs and no survivors were recruited through snowballing, reflecting a lack of social networking. Contacting survivors directly was much more successful, as all survivors were recruited this way.

### Either “Don’t Elaborate Further” or “Not Enough at All”: a Bifurcated Pattern of Information-Seeking Behaviors

Participants wanted more input and control over the information they received in their encounters with providers and the health care system. Information-seeking behaviors delineated into 2 groups. Some participants were not motivated to search for information, as it was “*an unhappy matter*” (S001). Receiving information did not mean feeling empowered or informed.

When I was diagnosed, I was constantly looking for information, but it was always unhappy. Always bad news. So I stopped. Even when the oncologist gives me the report, I tell them to keep it to “good news or bad news,” don’t elaborate further.

Participant S001

Other participants wanted to be informed about all options available and all aspects of their care, but felt that their information-seeking attempts were denied.

Eh… I feel like the doctors here give you relatively little information. Like, for example this [redacted], the information they give isn’t something I can get at the doctor’s. … I feel like it isn’t at all enough, not even quite enough. It’s not enough at all.

Participant S009

Participants who received information from their clinicians double-checked it with external resources, reflecting a sense of suspicion. However, external sources were also viewed with suspicion. Patients felt responsible for, but unable to parse the quality and accuracy of information they received from health care encounters and the system. Prestigious institutions were used as shorthand for quality, and videos were more digestible than text. Participants seeking information persevered in search of answers, and their perseverance while experiencing frustration carried them through these difficulties.

### “There’s Lots of Living in Between”: Chinese Canadian Survivors Feel Unsupported When Attempting to Address Their Needs

It’s kind of sad, isn’t it? That you have to be dying… before they do something. There’s lots of living in between that needs some kind of support… some kind of signal from beyond [that] somebody still cares about you. … I tend not to go so deep into this. I don’t want to spoil my day thinking that nobody cares.

Participant S005

Participants described a procedure-focused health care system that failed to deliver continuity of care and or provide satisfactory care unless they were in an advanced stage of cancer. These experiences reflected a lack of systemic resources for, and attention to the wide-ranging effects that PCa had on survivors’ quality of life. Unmet needs resulted in desires for better access to care, shorter wait times, continuous support from the point of diagnosis, and support with language access. Those who continued to be symptomatic felt that they were responsible for their own follow-up. Participants found decision-making support lacking and were frustrated with long waits. Few indicated that they asked for information about incorporating traditional Chinese medicine (TCM) into their care. While sexual dysfunction is included in validated surveys, it was not identified as a priority unmet need. Participants had expectations of what the system was supposed to provide. They saw the gap between their expectations and the care they received but felt unable to effect change in the system.

### “We Become a Lot Closer”: Relational and Familial Support Are Key Facilitators That Create a Sense of Comfort for Survivors

When encountering perceived systemic shortcomings, such as lack of translation services or communication accessibility, survivors turned toward familial support. Family members acted as facilitators during clinical interactions if they were able to do so. In concert with this type of relational support, higher formal education and fluency in English improved survivors’ ability to navigate the system.

…because I’m more educated, my thinking patterns – I can understand their pattern of thinking, so it’s easier for me to understand. If I describe – if my education level is lower, it wouldn’t be as easy for me to understand what they’re saying.

Participant S001

Participants sometimes struggled with placing their children in this role, as they knew that these duties were burdensome. They felt that this burden was heavier if they were not comfortable in their ability to interact with the health care system in English, as they had to ask their children to translate. Those without familial support attempted to endure and did not fully engage with external supports. However, they struggled more with system navigation, compounded if they also struggled with communicating in English. Overall, familial support was highlighted as key to comfort and safety. For participants in clinical trials, the ability to privately communicate with their trial manager was valued. This person was able to coordinate care and field questions. Participants grew to trust them, as this relationship created a similar feeling of support.

The middleman, the manager, the care is more personal. … every time, it’s them. It’s not like today it’s someone, another day someone else… we become a lot closer.

Participant S008

### “A Sense That He’s Still in the Loop”: Digital Health Technologies Must Strengthen Relationships to Provide Systemic Value

Similar to how participants bridged unmet needs with familial and relational support, their virtual care experiences and technology preferences all pointed to a need for connection. However, most felt that virtual care resulted in fractured care transactions, not stronger relationships, and the strength of participants’ connection with their clinician appeared correlated with how safe they felt when receiving care. Several used “better than nothing” to describe their impressions of telemedicine, as their experiences were generally limited to checking blood test results with their provider. Although telemedicine removed the logistical hassle, it did not change a lack of focus on improving quality of life for survivors.

Participants expressed that a virtual platform for survivorship management and follow-up care would be acceptable if they found it useful and were confident that they could use a potential app if support and sufficient time to adapt was provided. Participants used digital technology to connect to friends and family and to search for information. They did not consider themselves expert users or early adopters. Usability was stressed over aesthetics; they wanted straightforward interfaces, simple help documentation, and infrequent software updates.

Although the convenience of telemedicine was appealing, it did not fix existing problems. Participants desired a digital health tool that included scheduling support, information, care coordination, strengthened connection to their clinicians, and a personal health information repository. Those not comfortable communicating in English felt that a Chinese language tool would provide peace of mind by decreasing the burden on their children. Participants wanted digital health to reinforce care continuity. Many felt like they had been suddenly abandoned by their providers after treatment. Safety meant knowing that someone cared for them.

And someone you hope, in cyberspace is reading what you submitted and said, “Oh, maybe we should do a follow up, because… he just feels he’s only happy at a scale of three”, that kind of continued monitoring. Not every day, but from time to time. It gives the patient a sense that he’s still in the loop.

Participant S005

## Discussion

### Principal Findings and Implications

This study illustrated the structures influencing the experiences of Chinese Canadian PCa survivors in relation to the design of a culturally adapted patient follow-up care app. Several themes emerged among participants’ experiences with PCa follow-up care and virtual care. Emotions and difficulties were coped with by “eating bitterness” (吃苦) [58]. Care and support were highly relational and family-centered, and those without this support coped by eating bitterness. Finally, relationally connected care was emphasized as key to safety.

However, eating bitterness when facing systemic inequities individuates responsibility for structural issues. We reinforce research on unmet systemic needs within the broader survivor community, such as communication [59], health system [59], information support [60-62], and longitudinal supportive care needs [63]. Technology use reflected findings on older adult digital literacy and preferences [64-67]. TCM and sexual dysfunction were not explicitly spoken about, which may indicate that participants felt uncomfortable discussing these topics in their experiences with the health care system. The compounded isolating effects of the model minority stereotype and these norms show that while Chinese Canadian survivors may have similar needs to the wider survivor community, they feel unable to, ignored, or uncomfortable when voicing their needs. Systemic changes that materially support family involvement, accessibility for EAL speakers and patients with less formal education, and relationally connected, culturally safe care are needed to improve well-being. These changes are not specifically Chinese and may benefit other communities made vulnerable by settler colonialism.

Chinese Canadian survivors are seeking to strengthen their connections in a health care system that provides privacy and accessibility, protects relationality, and promotes transparency, accountability, and responsibility. Although age is a DDOH and survivors skew older, it is the self-confidence to make decisions that appears to be the top factor in health-related internet use for these survivors [68]. If Chinese Canadian survivors already feel unable to voice their needs within existing survivorship care processes, introducing digital technology into their care may strengthen this inability. Indeed, their perception of virtual care as “better than nothing” reflects the ways that the DDOH strengthens structural inequities. In the context of these inequities, we must critically reflect on the policies, systems, and practices that digital health tools are designed within to examine if we are designing places that allow for good care. The digital spread of hate indicates that it is designed with the protocols of settler colonialism [69]. Digital health tools are intimately influenced by the lands, relations, and communities with whom and where we live, and where and how these technologies are designed, developed, and used. Health services are location-specific, so digital health must also reflect these ties to be feasible.

These findings must be contextualized within the history of Chinese people in Canada and their experiences of extensive anti-Asian racism, which increased sharply during the COVID-19 pandemic [46,70]. Hate and mis- and disinformation, including anti-Asian racism, has been shown to be amplified by technology-mediated interactions [17]. From the first documented arrival of Chinese immigrants on “Canadian” shores in 1788, waves of successive immigration have created a vast and heterogeneous community [45,71]. This complexity further eludes a definition of Chinese Canadian culture for the purposes of health care delivery or cultural adaptation. While acknowledging the pragmatic applications of cultural adaptation frameworks, they generally use culturally sensitive or competent framings rather than cultural safety [72-74]. These framings may “museumize” and unintentionally problematize identity categories and culture as causes of ill health. This problematization echoes the long record of discrimination and stereotyping against Chinese Canadians and other communities made vulnerable in health care settings [59].

Instead, this study shows the utility and efficiency of taking a structural approach. If we prioritize good relations and cultural safety through a structural approach to digital health rather than simply digitizing or culturally adapting existing processes, we might begin to regenerate places of care through digital health design. By uncovering structures that create obstacles to good care, we identified relational, scalable design opportunities.

### Future Directions

This study contributes to the emerging field of “cultural” adaptations and design for chronic disease digital health innovations. Our intent was to identify opportunities for connection by taking a structural approach. Forthcoming work will integrate study findings into an adapted prototype of the *Ned* app according to UCD principles and the taxonomy developed by Spanhel et al [15]. This prototype will undergo usability testing by participants for evaluation and member-checking. To develop greater perspective and strengthen these findings, future work should examine the specific experiences of PCs providing survivorship care to reflect the importance of their role. Moreover, the stage of diagnosis and disease can affect the survivor’s quality of life issues; additional work examining how the severity of their PCa may impact survivors’ virtual follow-up care experiences is warranted.

### Strengths and Limitations

Findings should be considered with some limitations. Our sample does not fully represent the Chinese Canadian PCa community, as community characteristics make a representative sample difficult to fully reflect [71]. Further, participants may have been particularly interested in virtual care, which may result in selection bias. Thick description of our theoretical stance, setting and place, methods, and results are provided to enhance understanding. This study was completed in partnership with Chinese Canadian PCa survivors and in recognition of the specific place-based structures that affect their lives and care experiences. We think of and encourage the transferability of this research as how it might be made meaningful for other communities in places where they may be subject to similar constructs and patterns of oppression. This is relational validity, or how the roots and implications of this work are transferable to our specific lives, human relations, more-than-human relations, and the lands that we live with [75]. Finally, this study does not include the provider perspective, though *Ned* was developed with clinicians who provide follow-up care for patients from this community.

### Conclusions

We locate structures that influence the desires and challenges within Chinese Canadian PCa survivors’ stories of virtual and follow-up care. They feel that they eat bitterness when coping with PCa and must rely on the support of family and relations when receiving follow-up care. However, eating bitterness when facing systemic inequities individuates responsibility for structural issues. Systemic changes that materially support family involvement, accessibility for EAL speakers and patients with less formal education and relationally connected, culturally safe care are needed. Grounding digital health in critical decolonial and social justice stances is key to designing places for good care. Instead of museumizing culture through cultural adaptation, a grounded and structural approach that follows Indigenous research and Black feminist thought on the necessity of multiple perspectives, vitality of land, and relationality of all provides entry points for collective change through digital health design.

## Appendix

Multimedia Appendix 1 Semistructured interview guide for survivor participants.

Multimedia Appendix 2 Semistructured interview guide for partner-caregiver (PC) participants.

Multimedia Appendix 3 Demographic characteristics of survivor and partner-caregivers (PC) participants in table form.

## Abbreviations

CBPR: community-based participatory research
COREQ: Consolidated Criteria for Reporting Qualitative Research
DDOH: digital determinants of health
EAL: English as an additional language
GVRD: Greater Vancouver Regional District
Ned: no evidence of disease
PC: partner-caregiver
PCa: prostate cancer
REDCap: Research Electronic Data Capture
SDOH: social determinants of health
TCM: traditional Chinese medicine
UCD: user-centered design

## Data Availability

The data sets generated and analyzed during this study are available from the corresponding author upon reasonable request.
