# Supplementary material for: Honoring the Care Experiences of Chinese Canadian Survivors of Prostate Cancer to Cultivate Cultural Safety and Relationality in Digital Health: Exploratory-Descriptive Qualitative Study
Source: J Med Internet Res. 2023 Dec 28;25:e49349. doi: 10.2196/49349 (PMC10784982; doi:10.2196/49349)
Supplement: Multimedia Appendix 1 [file jmir_v25i1e49349_app1.docx]

## Semi-Structured Interview Guide - Survivor

Thank you for taking the time to speak to us as someone with lived experience related to prostate cancer. We are researchers from the University of Toronto. The objective of this study is to understand how virtual care can support Chinese Canadian prostate cancer patients in the survivorship phase of their cancer trajectory. As part of this research, we are conducting two phases of interviews with survivors and caregivers of prostate cancer survivors. This interview is part of the first phase.

The purpose of the questions is simply to guide the conversation. However, because the goal is to understand your experiences, you should feel free to talk about anything you think might be important. You can also ask me to repeat or rephrase a question if you don’t understand. Finally, you also have the choice to skip a question or end the interview at any time. There are no right or wrong answers, as we are interested in learning more about your opinions and experiences.

Your name or other identifying information will be kept confidential. Your decision to participate, withdraw, or skip questions will have no effect on your current or future care. We will likely spend 60 minutes together, but this can be longer or shorter depending on your preference. Do you have any questions before we begin? In the letter of information, it is noted that I will audio or video record the session. Is it still okay if I audio or video record the session? Do you agree to take part in this interview? [*wait for response*] Thank you and thank you for your participation today. Okay, I will start recording now [*researcher turns recording on*].

1. To start us off, I would like to learn about you. Could you tell me a little bit about yourself and about your background?
   [Probes: Where do you live, and how long have you lived there? Do you live with your family? Who are your family members? What do you do for work if you work? Do you have any hobbies you like to do?]
2. Can you tell us about your experiences with prostate cancer follow-up care?
   [Probes: Could you tell us when you were first diagnosed? What kind of treatment did you receive? What happened after you finished your last treatment?]
3. Tell me about the information you receive when you visit your doctor for follow-up care. Can you give me an example? Do you usually see the doctor who treated you, or someone else (i.e. resident doctor, medical fellow, medical student, nurse)?
4. Can you walk me through a typical follow-up appointment?
5. How do you feel about the follow-up care you receive?
6. Do you usually attend your appointments by yourself? If you usually have someone attend your appointments with you, what is your relationship to this person? What do they usually do at these appointments?
7. Do you feel comfortable or safe when you have a follow-up appointment? If you do, what makes you feel that way? If you do not, could you explain why?
8. In reflecting on your experience with prostate cancer follow-up care, is there anything you like about it? Is there anything you would like to be different?
   [Probes: Did you understand the information you were given? What level of detail did they give you? Was it enough information?]
9. Are there any other sources where you have gone to find information about prostate cancer follow-up care? What has made these sources useful to you? Have you experienced any issues with accessing or understanding this information?
10. Has COVID-19 changed the way that your doctor provides your prostate cancer follow-up care? How has it changed? How do you feel about these changes?
11. Do you have any other thoughts about your prostate cancer follow-up care that you think it would be important for us to know to improve the experience of other survivors in the future?

Our research team is also interested in learning about your experiences and perceptions of virtual care. Virtual care can be defined as any interaction between you and your circle of care, occurring remotely, using any forms of communication or information technologies with the aim of facilitating your care. Virtual care includes applications such as telemedicine visits with your doctors, or telemonitoring a chronic condition through a phone application.

1. Are you comfortable using computers or smartphones to access information? What kind of applications do you use if you use them?
2. Have you had any medical appointments through virtual care? If you have, what was your experience like?
3. Is there anything you wish you had known or could have been told before you started using virtual care? If you are comfortable doing so, please explain.
4. Have you asked for help when accessing virtual care? What kind of help have you asked for, and where did you get it? Was any of this help particularly helpful to you?
5. How do you feel about virtual care? Is it something that you feel comfortable or safe using? If you are comfortable doing so, can you explain why?
6. In reflecting on your experience with virtual care, is there anything you like about it? Is there anything you would like to be different?

We would like to ask your opinion about the following. We are proposing a model of care where you will be asked to complete your follow-up care tasks, such as your blood test and a health survey, through a web application. Your doctor will review your test and survey results. Then, they will send you a Doctor’s Note through the application that will tell you if everything is OK, or if additional follow-up is needed (through the phone or in-person if necessary). In this model of care, you will not be seeing or speaking to your doctor in real-time. This is called an “asynchronous” model of care.

1. Would you be comfortable with this model of care as described?
2. Could you explain why you would or would not be comfortable? If you are not, is there anything that you think would be helpful in making you more comfortable with this model of care?

Finally, we would like to return to the topic of prostate cancer follow-up care.

1. Have you talked about or shared your experiences with prostate cancer or prostate cancer follow-up care with your family, friends, or fellow survivors? Do you think that they might be interested in participating in this study? Would you be comfortable providing their names and contact information so we could ask if they might be interested in participating?

Thank you so much for sharing your experiences. We appreciate your time, willingness, and openness in talking with us today. Do you have any questions about our interview today and/or this study? In the next 2 weeks, we will be sending you an honorarium in the amount of $50.00 per hour in appreciation of your time. It is our hope that this study will be helpful to inform us on how best to provide and design care for patients like you in the future.

After we end our call, I will send the gift card to your email (confirm email address) along with a receipt form to confirm that you received the honorarium. Please fill out this receipt with your name and date and send it back to me. I will also be sending you a completed copy of the consent form, with my signature, for your records. If you have any questions after the study, please don’t hesitate to reach out by phone or email. Thank you for attending this session!
