# Supplementary material for: Honoring the Care Experiences of Chinese Canadian Survivors of Prostate Cancer to Cultivate Cultural Safety and Relationality in Digital Health: Exploratory-Descriptive Qualitative Study
Source: J Med Internet Res. 2023 Dec 28;25:e49349. doi: 10.2196/49349 (PMC10784982; doi:10.2196/49349)
Supplement: Multimedia Appendix 2 [file jmir_v25i1e49349_app2.docx]

## Semi-Structured Interview Guide - PC

Thank you for taking the time to speak to us about your experience caring for someone living with prostate cancer. We are researchers from the University of Toronto. The objective of this study is to understand how virtual care can support Chinese Canadian prostate cancer patients in the survivorship phase of their cancer trajectory. As part of this research, we are conducting two phases of interviews with survivors and caregivers of prostate cancer survivors. This interview is part of the first phase.

The purpose of the questions is simply to guide the conversation. However, because the goal is to understand your experiences, you should feel free to talk about anything you think might be important. You can also ask me to repeat or rephrase a question if you don’t understand. Finally, you also have the choice to skip a question or end the interview at any time. There are no right or wrong answers, as we are interested in learning more about your opinions and experiences.

The questions for this interview are divided into two sections. First, I will ask you some general questions about your lived experience as a caregiver. Then, I will ask questions about your opinion about your experience with virtual care. Throughout the interview, please try to avoid using names when talking about the person you provide care to or their healthcare providers - but don’t worry too much if you say their names because we will redact it in the transcript.

Your name or other identifying information will be kept confidential. Your decision to participate, withdraw, or skip questions will have no effect on your current or future care. We will likely spend 60 minutes together, but this can be longer or shorter depending on your preference. Do you have any questions before we begin? In the letter of information, it is noted that I will audio or video record the session. Is it still okay if I audio or video record the session? Do you agree to take part in this interview? [*wait for response*] Thank you and thank you for your participation today. Okay, I will start recording now [*researcher turns recording on*].

1. To start us off, I would like to learn about you. Could you tell me a little bit about yourself and about your background?
   [Probes: Where do you live, and how long have you lived there? Do you live with your family? Who are your family members? What do you do for work if you work? Do you have any hobbies you like to do?]
2. Can you tell us about your experiences with caregiving for someone who is a prostate cancer survivor?
   [Probes: Could you tell us when your partner was first diagnosed? What kind of treatment did they receive, and what was it like? What happened after they finished their most recent treatment?]
3. Do you usually attend your partner’s prostate cancer follow-up care appointments?
   1. *If yes:* Can you walk me through a typical follow-up appointment? What information did you receive as part of that appointment?
   2. *If no:* Does your partner tell you about their follow-up care appointments? What information do they tell you, if they tell you anything?
4. How do you feel about the follow-up care your partner receives?
5. Does the follow-up care your partner receives feel safe or adequate to you? If it does, what makes it feel that way? If it does not, could you explain why?
6. In reflecting on your experience with your partner’s prostate cancer follow-up care, is there anything you like about it? Is there anything you would like to be different?
   [Probes: Do you feel like the information they are given can be understood? What level of detail did they give you? Was it enough information?]
7. Are there any other sources where you have gone to find information about prostate cancer follow-up care? What has made these sources useful to you? Have you experienced any issues with accessing or understanding this information?
8. Has COVID-19 changed the way that your partner’s doctor provides your prostate cancer follow-up care? How has it changed? How do you feel about these changes?
9. Do you have any other thoughts about your partner’s prostate cancer follow-up care that you think it would be important for us to know to improve the experience of other survivors in the future?

Our research team is also interested in learning about your experiences and perceptions of virtual care. Virtual care can be defined as any interaction between you and your circle of care, occurring remotely, using any forms of communication or information technologies with the aim of facilitating your care. Virtual care includes applications such as telemedicine visits with your doctors, or telemonitoring a chronic condition (such as diabetes, hypertension, or heart failure) through an application on your phone.

1. Are you comfortable using computers or smartphones to access information? What kind of applications do you use if you use them?
2. Have you had any medical appointments through virtual care? If you have, what was your experience like?
3. Do you assist your partner with accessing virtual care?
4. Is there anything you wish you had known or could have been told before you or your partner started using virtual care? If you are comfortable explaining, please do so.
5. Have you or your partner asked for help when accessing virtual care? What kind of help have you asked for, and where did you get it? Was any of this particularly helpful to you?
6. How do you feel about virtual care? Is it something that you feel comfortable or safe using? Does your partner feel comfortable or safe using virtual care?
7. In reflecting on your or your partner’s experience with virtual care, is there anything you like about it? Is there anything you would like to be different?

We would like to ask your opinion about the following. We are proposing a model of care where the survivor, your partner, will be asked to complete their follow-up care tasks, such as their blood test and a health survey, through a web application. Their doctor will review their test and survey results. Then, the doctor will send them a Doctor’s Note through the application that will tell them if everything is OK, or if additional follow-up is needed (through the phone or in-person if necessary). In this model of care, they will not be seeing or speaking to their doctor in real-time. This is called an “asynchronous” model of care.

1. Do you think they would be comfortable with this model of care as described? Would you be comfortable with this model of care?
2. Could you explain why either of you might or might not be comfortable with this model of care? Is there anything that you think would be helpful in making either of you more comfortable with this model?

Finally, we would like to return to the topic of prostate cancer follow-up care.

1. Have you talked about or shared your experiences as a caregiver for someone with prostate cancer with your family or friends? Do you think that they might be interested in participating in this study? Would you be comfortable providing their names and contact information so we could ask if they might be interested in participating?

Thank you so much for sharing your experiences. We appreciate your time, willingness, and openness in talking with us today. Do you have any questions about our interview today and/or this study? In the next 2 weeks, we will be sending you an honorarium in the amount of $50.00 per hour in appreciation of your time. It is our hope that this study will be helpful to inform us on how best to provide and design care for patients like you in the future.

After we end our call, I will send the gift card to your email [confirm email address] along with a receipt form to confirm that you received the honorarium. Please fill out this receipt with your name and date and send it back to me. I will also be sending you a completed copy of the consent form, with my signature, for your records. If you have any questions after the study, please don’t hesitate to reach out by phone or email. Thank you for attending this session!
