# Supplementary material for: Honoring the Care Experiences of Chinese Canadian Survivors of Prostate Cancer to Cultivate Cultural Safety and Relationality in Digital Health: Exploratory-Descriptive Qualitative Study
Source: J Med Internet Res. 2023 Dec 28;25:e49349. doi: 10.2196/49349 (PMC10784982; doi:10.2196/49349)
Supplement: Multimedia Appendix 3 [file jmir_v25i1e49349_app3.docx]

| Table S1 |  |  |
| --- | --- | --- |
| Demographic characteristics of survivor and partner-caregiver participants (N=14) |  |  |
|  |  |  |
| Individual-level variable: |  |  |
| Gender, n (%) | n | % |
| Female | 2 | 14% |
| Male | 12 | 86% |
|  |  |  |
| Age, years (missing values) |  |  |
| Values | 13 | 93% |
| Values, mean | 66.077 |  |
|  |  |  |
| Preferred identification |  |  |
| Chinese Canadian | 7 | 50% |
| Hong Kong Chinese | 4 | 29% |
| Other | 3 | 21% |
|  |  |  |
| Place of Origin |  |  |
| China | 4 | 29% |
| Hong Kong | 7 | 50% |
| Other | 3 | 21% |
|  |  |  |
| Year of Immigration |  |  |
| 1960-1969 | 1 | 7% |
| 1970-1979 | 2 | 14% |
| 1980-1989 | 3 | 21% |
| 1990-1999 | 7 | 50% |
| 2000 and after | 1 | 7% |
|  |  |  |
| Do you speak English as an additional language? |  |  |
| Yes | 13 | 93% |
| No | 0 | 0 |
| N/A | 1 | 7% |
|  |  |  |
| What is your highest completed level of education? |  |  |
| High school diploma | 1 | 7% |
| College trade or technical diploma | 6 | 43% |
| University undergraduate degree (e.g. BA, B.Eng) | 3 | 21% |
| University professional designation (e.g. MD, MBA) | 1 | 7% |
| University postgraduate degree (e.g. MSc, PhD) | 3 | 21% |
|  |  |  |
| What is your current living arrangement? |  |  |
| Living with a partner and/or family member(s) | 12 | 86% |
| Living alone | 2 | 14% |
|  |  |  |
| What type of area do you currently live in? |  |  |
| Urban (in a city) | 13 | 93% |
| Suburban (in a community adjacent to a city) | 1 | 7% |
|  |  |  |
| What is your current marital status? |  |  |
| Married/common-law union | 12 | 86% |
| Separated or divorced | 2 | 14% |
|  |  |  |
| What is your employment status? |  |  |
| Work full time | 2 | 14% |
| Work part time | 3 | 21% |
| Retired | 9 | 64% |
|  |  |  |
| What is your annual household income? |  |  |
| <$15,000 | 1 | 7% |
| $15,000 to $29,999 | 2 | 14% |
| $30,000 to $49,999 | 5 | 36% |
| $50,000 to $74,999 | 1 | 7% |
| $75,000 to $100,000 | 2 | 14% |
| >$100,000 | 1 | 7% |
| I prefer not to answer this question. | 2 | 14% |
|  |  |  |
| What is your preferred device? |  |  |
| Desktop / laptop (e.g. PC, Macbook, ThinkPad) | 7 | 50% |
| Smartphone (e.g. iPhone, Samsung Galaxy) | 7 | 50% |
| Tablet (e.g. Windows Surface, Google Chromebook, iPad) | 0 | 0% |
|  |  |  |
| How comfortable are you with your preferred device? |  |  |
| Very comfortable | 2 | 14% |
| Comfortable | 10 | 71% |
| Somewhat comfortable | 2 | 14% |
|  |  |  |
| Please estimate how often you use your preferred device: |  |  |
| Frequently (a few times a day) | 13 | 93% |
| N/A | 1 | 7% |
|  |  |  |
| App use: |  |  |
| Communication (e.g. Phone calls, texts, video calls, emails) | 12 | 86% |
| Information (e.g. Maps, news, weather) | 11 | 79% |
| Scheduling (e.g. To-do lists, appointments) | 9 | 64% |
| Information storage (e.g. Contacts) | 9 | 64% |
| Leisure (e.g. Instagram, Angry Birds, YouTube) | 12 | 86% |
| Health (e.g. Step counter, calorie management, medication management) | 6 | 43% |
| Other (photography) | 2 | 14% |
|  |  |  |
| How do you access digital services? |  |  |
| Desktop / laptop (e.g. Macbook, ThinkPad) | 9 | 64% |
| Smartphone (e.g. iPhone, Samsung Galaxy) | 9 | 64% |
| Tablet (e.g. iPad) | 6 | 43% |
| All of the above | 7 | 50% |
| I do not use any digital services | 0 | 0% |
|  |  |  |
| How many mobile health and wellness apps (e.g. Fitbit, Maple, etc.) do you use on your smartphone? |  |  |
| None | 8 | 57% |
| 2-4 | 5 | 36% |
| 4-8 | 1 | 7% |
|  |  |  |
